# A Novel GaN:C Millimeter-Wave HEMT with AlGaN Electron-Blocking Layer

**DOI:** 10.3390/ma15030703

**Published:** 2022-01-18

**Authors:** You-Chen Weng, Yueh-Chin Lin, Heng-Tung Hsu, Min-Lu Kao, Hsuan-Yao Huang, Daisuke Ueda, Minh-Thien-Huu Ha, Chih-Yi Yang, Jer-Shen Maa, Edward-Yi Chang, Chang-Fu Dee

**Affiliations:** 1 College of Photonics, National Yang Ming Chiao Tung University, Tainan 71150, Taiwan; ycweng.cop00g@nctu.edu.tw (Y.-C.W.); jershenmaa@mail.nctu.edu.tw (J.-S.M.); 2 Department of Materials Science and Engineering, National Yang Ming Chiao Tung University, Hsinchu 30010, Taiwan; nctulin@yahoo.com.tw (Y.-C.L.); b9904133@gmail.com (M.-L.K.); thienhuu84@gmail.com (M.-T.-H.H.); 3 International College of Semiconductor Technology, National Yang Ming Chiao Tung University, Hsinchu 30010, Taiwan; hthsu@nctu.edu.tw (H.-T.H.); axiarll44@gmail.com (H.-Y.H.); daisuke@ieee.org (D.U.); abx50131@gmail.com (C.-Y.Y.); 4 Department of Electronics Engineering, National Yang Ming Chiao Tung University, Hsinchu 30010, Taiwan; 5 Institute of Microengineering and Nanoelectronics (IMEN), University Kebangsaan Malaysia, Level 4, Research Complex, Bangi 43600, Malaysia; cfdee@ukm.edu.my

**Keywords:** GaN, HEMT, electron-blocking layer

## Abstract

An AlGaN/GaN/Si high electron mobility transistor (HEMT) using a GaN:C buffer with a 2 nm AlGaN electron-blocking layer (EBL) is investigated for the first time for millimeter-wave applications. Compared with the double heterostructure field effect transistor (DHFET), the AlGaN/GaN HEMT with the GaN:C/EBL buffer has a lower vertical leakage, higher thermal stability, and better RF performance. In addition, AlGaN EBL can prevent carbon-related traps from GaN:C and improve electron confinement in 2DEG during high-frequency operation. Finally, a P_out_ of 31.2 dBm with PAE of 21.7% were measured at 28 GHz at 28 V. These results demonstrated the great potential of HEMTs using GaN:C with AlGaN EBL epitaxy technology for millimeter-wave applications.

## 1. Introduction

Due to the wide bandgap and high mobility nature of the GaN material, AlGaN/GaN HEMTs have become one of the most popular devices for high-frequency and high-power applications, including 5G applications, in recent years. In the past decade, AlGaN/GaN HEMTs on SiC have shown efficiency over 40% [1,2,3], and a high output power density of 8.2 W/mm at Ka band [4]. However, the fabrication cost of AlGaN/GaN HEMTs on SiC is very high. Recently, AlGaN/GaN HEMTs on Si substrate for Ka band applications have become popular [5], since GaN-on-Si substrate technology has the benefit of fabricating devices on large wafers (up to 8 inch) with silicon-compatible processes [6,7] to reduce manufacturing costs as compared with GaN-on-SiC technology.

Nevertheless, RF loss caused by the buffer layers for AlGaN/GaN HEMTs on Si substrate results in a lower output power when the device is used for millimeter-wave applications. Several issues have to be addressed for the buffer layer’s epitaxy for RF loss improvement, including AlN/Si interface issues, and different kinds of buffers, including the buffer (thickness usually around 0.5~2 μm) and back-barrier layer (thickness usually around 0.5~1 μm) in the epitaxial structure. For AlN/Si interface properties, parasitic channel formation at the Al/Si interface [8] is usually encountered due to the diffusion of Al and Ga atoms into Si substrate. Furthermore, a buffer was grown to balance the lattice and thermal mismatch between GaN and Si for stress control, three major types of buffer structures have been studied in the past [9], namely, the step AlGaN buffer, the low-temperature interlayer (LT-interlayer) buffer, and the (Al)GaN/AlN superlattice buffer. The step AlGaN buffer has multi-AlGaN layers, each layer typically around 100 to 300 nm thick, and each AlGaN layer with decreasing Al%. The LT-interlayer buffer includes some transitions layers (e.g., AlGaN, carbon doped, and iron doped), and the thin LT-Al(Ga)N layer was used to introduce compressive stress. The superlattice buffer uses a thin layer of (Al)GaN/AlN to build up compressive stress. However, some defects still form during the growth of the thick layer. Finally, with the back-barrier layer being the first layer under the undoped GaN channel, different doping profiles may act as the acceptors or donors which may degrade the channel conductivity, resulting in current collapse [10,11,12,13].

Recently, DHFET on Si substrate was investigated for RF applications [14]. DHFET usually adopts a thick AlGaN as the back-barrier (thickness usually over 1μm) with 4~10% Al content to achieve high breakdown voltage due to better electron confinement with low trapping effects [5,15,16]. Using the GaN:C buffer as back-barrier is widely used in high power switching applications [17,18] with low memory effect [19]. However, the transfer of hot carriers from the channel to the GaN:C buffer occurs at high drain bias levels, which causes device degradations for millimeter-wave applications [11,12,13,14]. Some papers report positive results using the AlGaN back-barrier combined with the GaN:C buffer to enhance the channel conductivity of the device, and to reduce the current collapse effect [20,21,22,23]. Moreover, according to [24], W. Liu et al. reported when 10 < Al% < 90, poor thermal conductivity of the AlGaN layer will cause a self-heating effect in the device, thus increasing the channel temperature during the device operation. In addition, the device’s current collapse for different types of buffers was also evaluated [9], and this showed that a low current collapse effect can be achieved by using a superlattice as a buffer.

In this paper, an AlGaN/GaN HEMT with a GaN:C buffer, combined with low Al content Al_0.05_Ga_0.95_N EBL and a AlN/GaN superlattice buffer was grown on Si substrate. The result is compared to the AlGaN/GaN/AlGaN DHFET on Si substrate for RF applications.

## 2. Materials and Methods

**Figure 1:**
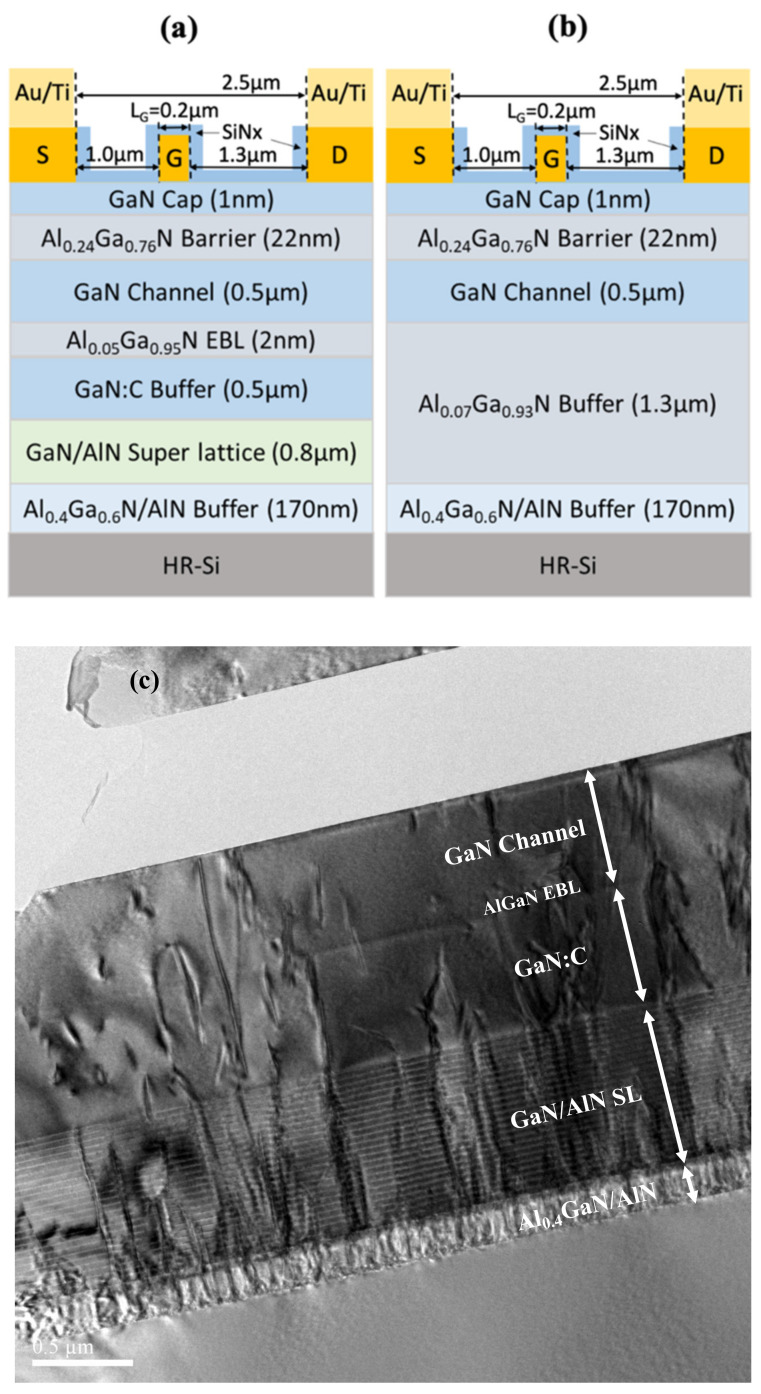

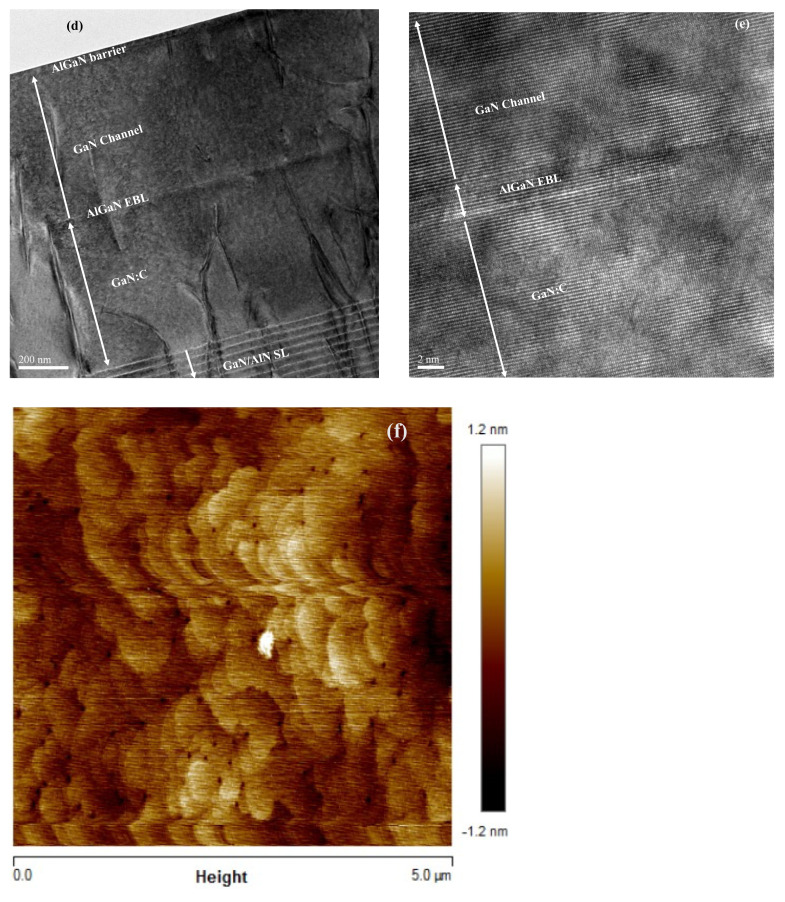
(**a**) The device structure of the GaN:C buffer AlGaN/GaN HEMT with AlGaN EBL; (**b**) the device structure of the AlGaN/GaN/AlGaN DHFET; (**c**) cross-section of the TEM image of the AlGaN/GaN HEMT with GaN:C buffer with AlGaN EB; (**d**) the TEM image of the GaN channel/AlGaN EBL/GaN:C, (**e**) The TEM image of the AlGaN EBL; (**f**) surface morphology of the GaN:C buffer AlGaN/GaN HEMT with AlGaN EBL.

The AlGaN/GaN HEMTs structure with the GaN:C/EBL buffer were grown by the Veeco Propel Metal Organic Vapor Deposition (MOCVD) system on high resistivity 6-inch Si (111) substrate (R = 3000 ohm-cm). From the bottom to the top, the epitaxial layer structure of the device consisted of a 120 nm AlN seed layer, a 50 nm Al_0.4_Ga_0.6_N buffer layer, an 800 nm AlN/GaN super-lattice buffer layer, a 500 nm GaN:C back-barrier layer, a 2 nm Al_0.05_Ga_0.95_N electron blocking layer, a 500 nm unintentionally doped (UID) GaN channel layer, a 22 nm Al_0.24_Ga_0.76_N barrier layer, and a 1nm GaN cap layer; the structure is as shown in Figure 1a. The room-temperature electron mobility of 1700 cm^2^/V•s and a sheet carrier density of 8.5 × 10^12^/cm^2^ were achieved for the AlGaN/GaN HEMT device structure with the GaN:C/EBL buffer. The structure of DHFETs on Si substrate was shown in Figure 1b, and the room-temperature electron mobility of 1750 cm^2^/V•s and a sheet carrier density of 8.0 × 10^12^/cm^2^ were measured. Figure 1c shows the cross-sectional transmission electron microscopy (TEM) images of the GaN:C/EBL buffer HEMT device; the interfaces of the buffer were quite smooth, as shown in this figure. Figure 1d shows the sharp and smooth interface between the GaN channel, AlGaN EBL, and the GaN:C back-barrier, and the threading dislocations in the GaN:C were effectively bent by the AlGaN EBL. Additionally, this is first time using a TEM image to verify the AlGaN EBL thickness in a AlGaN/GaN HEMT epitaxy structure. The 2 nm AlGaN EBL is shown in Figure 1e, Figure 1f shows the root mean square roughness of the AlGaN/GaN HEMTs structure with the GaN:C/EBL buffer surface morphology measured by the Atomic Force Microscope (AFM). The surface roughness was 0.264 nm.

The HEMT device process consisted of four major steps: ohmic contact formation, ion implantation isolation, gate formation, and interconnecting formation. First, the fabrication process starts with ohmic contact formation. Ti/Al/Ni/Au ohmic metal was deposited by E-gun evaporation, followed by the lift-off process, and then annealed at 850 °C for 30 s in an N_2_ ambient environment by a rapid thermal annealing system (RTA). After that, an ion implantation isolation process using 3 × 10^13^ B11^+^ with 190 keV ion implantation energy was performed to define the active region. For device gate formation, a 100 nm SiNx passivation layer was deposited using plasma-enhanced chemical vapor deposition (PECVD). The gate stem was fabricated by E-beam lithography and SiNx etching by was performed with ICP, and the gate metal Ni/Au was deposited by E-gun evaporator, followed by the lift-off process. The gate length was around 200 nm in this study. Finally, a 100 nm SiN_x_ layer was deposited for passivation with nitride via the open-to-the-device pad region; finally, the thick metal stacks with Ti (30 nm)/Au (1000 nm) were deposited by E-gun evaporator.

## 3. Results and Discussion

**Figure 2:**
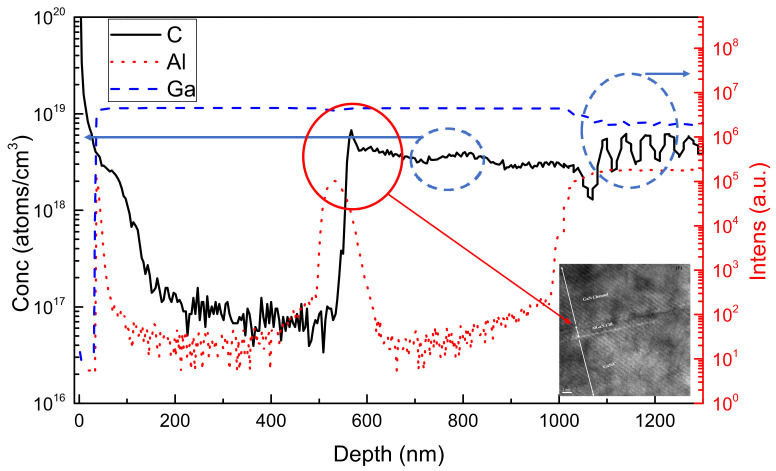
SIMS depth profiles of C, Ga, Al for the AlGaN/GaN HEMT with GaN:C and AlGaN EBL buffer, the insert shows AlGaN EBL between GaN:C and UID GaN channel.

To verify the material compositions of each layer, the secondary ion mass spectrometry (SIMS) analysis was performed. The composition profiles of the AlGaN/GaN HEMT device structure with AlGaN EBL are shown in Figure 2. From the depth profiles, C and Al atoms were observed at a distance of 500 nm below the surface, showing evidence that an amount of Al atoms existed between the GaN:C and UID GaN channel. It was found that inserting an AlGaN EBL between the UID GaN channel and the GaN:C buffer can be used to prevent carbon-related traps from the GaN:C buffer; however, the Ga and Al signals were used only for quality analysis, not for quantitative analysis. From Figure 2 and the insert of Figure 2, a 2 nm thick AlGaN EBL can be clearly observed from the TEM image.

**Figure 3:**
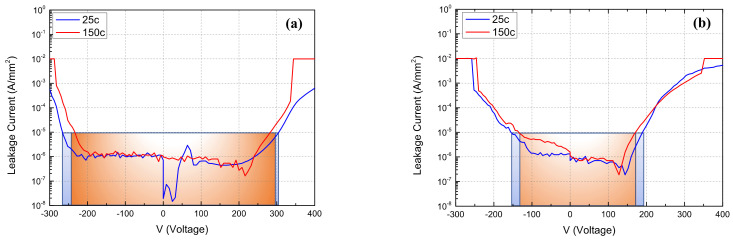
Vertical buffer leakage currents under forward and reverse bias (@Temperature: 25 °C and 150 °C) for (**a**) the GaN:C/EBL buffer device, and (**b**) the DHFET.

Figure 3 shows the temperature-dependent forward (AlGaN/GaN surface to Si substrate) and reverse (Si substrate to AlGaN/GaN surface) vertical leakage currents between the GaN channel and the Si substrate. The forward bias was swept up from 0 V to 400 V (step is 1 V), the reverse bias from 0 V to −300 V (step is −1 V), with temperature ranging from 25 °C (room temperature) to 150 °C. The vertical breakdown voltage (V_bd_) was defined at the leakage current of 10^−5^ A/mm^2^. The V_bd_ of the device with GaN:C/EBL buffer decreased from 305 to 280 V (9% decrease) at forward bias, and −270 to −230 V (15% decrease) at reverse bias, as shown in Figure 3a. However, asymmetric characteristics were observed in reverse and forward characteristics in Figure 3b. The Si substrate formed a barrier at AlN/Si interface, but multi-AlGaN buffer could not prevent the electrons from injecting into the buffer layer. The V_bd_ of the DHFET decreased from 195 to 170 V (13% decrease) at forward bias, and −160 to −130 V (19% decrease) at reverse bias, as shown in Figure 3b. Thus, the device with the GaN:C/EBL buffer had a higher V_bd_ field strength (1.5 times higher than DHFET) and higher thermal stability at high temperatures.

**Figure 4:**
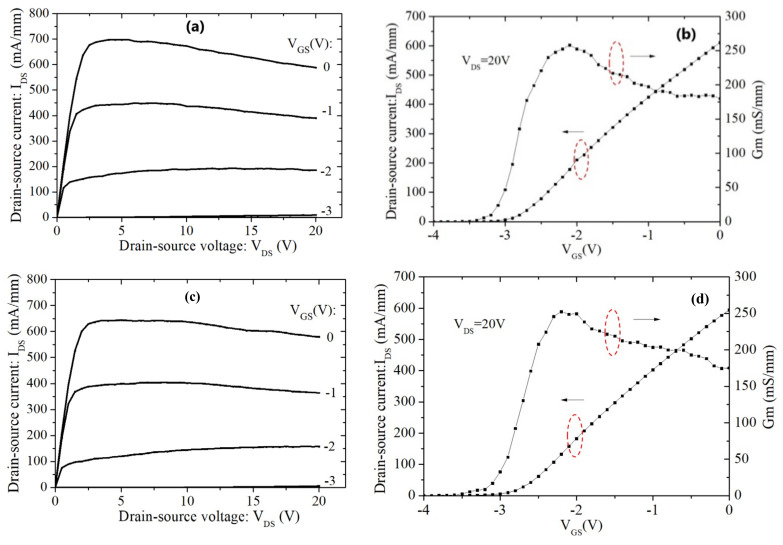
(**a**) I_DS_-V_DS_, and (**b**) I_DS_/G_m_-V_GS_ characteristics of the 2 × 50 μm GaN:C/EBL buffer device. (**c**) I_DS_-V_DS_, and (**d**) I_DS_/G_m_-V_GS_ characteristics of the 2 × 50 μm DHFETs.

**Figure 5:**
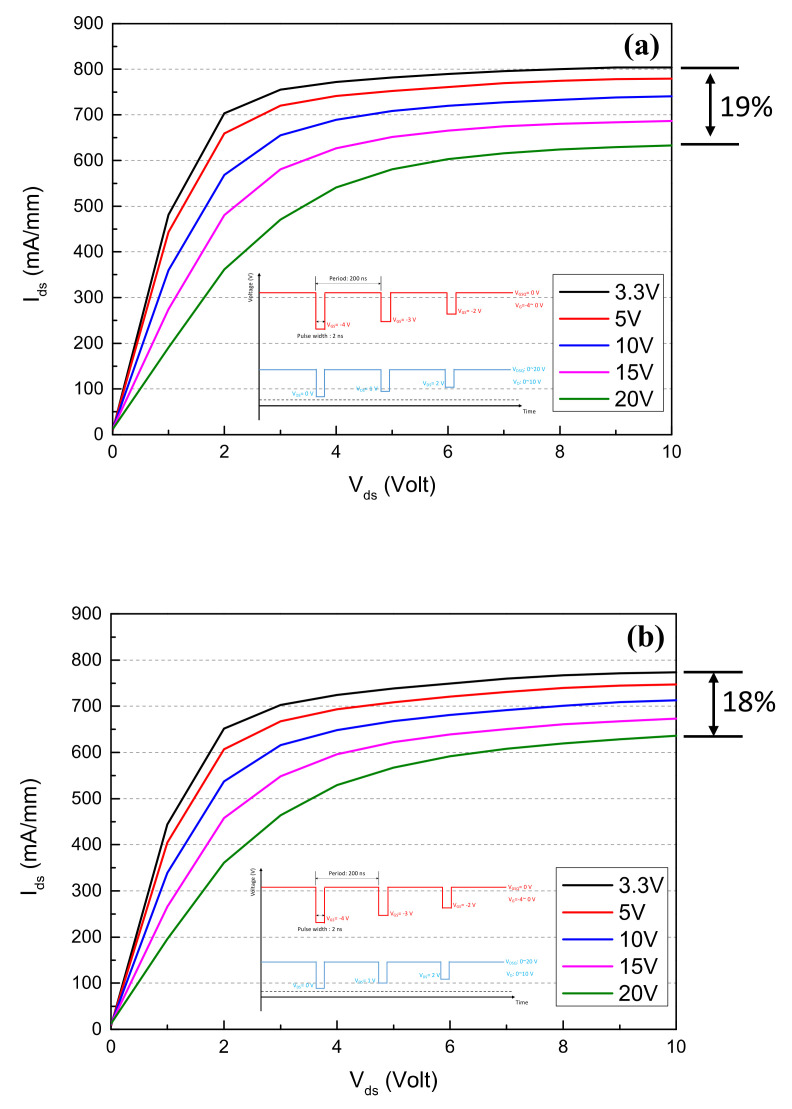

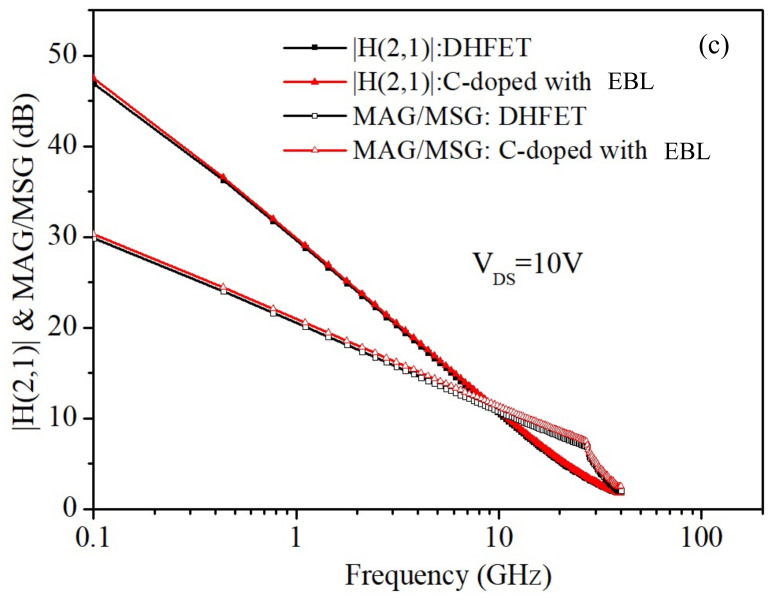
Pulsed I-V characteristics of the device with different buffers: the data show the drain lag under different drain biases (pulse width 200 ns, duty 1%); (**a**) the 2 × 50 μm GaN:C/EBL buffer device; (**b**) the 2 × 50 μm DHFETs; (**c**) MSG/MAG gain of the GaN:C/EBL buffer device and the DHFET (device size 8 × 50 μm).

Figure 4a,b show the DC characteristics including the current–voltage (I_DS_-V_GS_) curve and the transfer curve (G_m_-V_GS_) of the 2 × 50 μm GaN:C/EBL buffer device. The GaN:C/EBL buffer device exhibits an I_DS_ of 611 (mA/mm) and a maximum G_m_ of 258 mS/mm at V_DS_ = 20 V. Compared with the GaN:C/EBL buffer device, the DHFET showed a lower I_DS_ of 594 mA/mm and a lower maximum G_m_ of 250 mS/mm at V_DS_ = 20 V, as shown in Figure 4c,d, due to lower sheet carrier density for the DHFET. Pulsed I_DS_-V_DS_ characteristics were extracted from the off-state with quiescent bias (VGSQ) of −4 V to on-state at 0 V in 200 ns with 1% duty as shown in Figure 5a,b. As observed, current collapse occurred in both samples. The quiescent drain bias (VDSQ) was swept from 3.3 to 20 V (3.3, 5, 10, 15, 20 V). The dynamic on-resistance increased from 2.1 to 5.2 Ω mm and increased from 2.3 to 5.2 Ω·mm when VDSQ varied from 3.3 to 20 V for the GaN:C/EBL buffer device and the DHFET, respectively. From the pulsed IV results, the device with the GaN:C/EBL buffer had similar results with the DHFET device after the drain lag test, because the electrical field shielding effect of the AlGaN EBL, which effectively suppressed the capture of electrons in the channel by carbon-induced acceptor, trapped them in the GaN:C buffer [21].

Figure 5c shows the comparison of the measured small-signal maximum available (stable) gain (MAG/MSG) and current gain (H21) for the 8 × 50 μm device of the device with GaN:C/EBL buffer and DHFET. The measurements were performed using vector network analyzer on the on-wafer probing system up to 40 GHz. The GaN:C/EBL buffer device showed a consistent improvement of about 1 dB in MAG/MSG up to 30 GHz, evidencing that the AlGaN EBL could reduce the current collapse effect observed for the device using only the GaN:C buffer.

**Figure 6:**
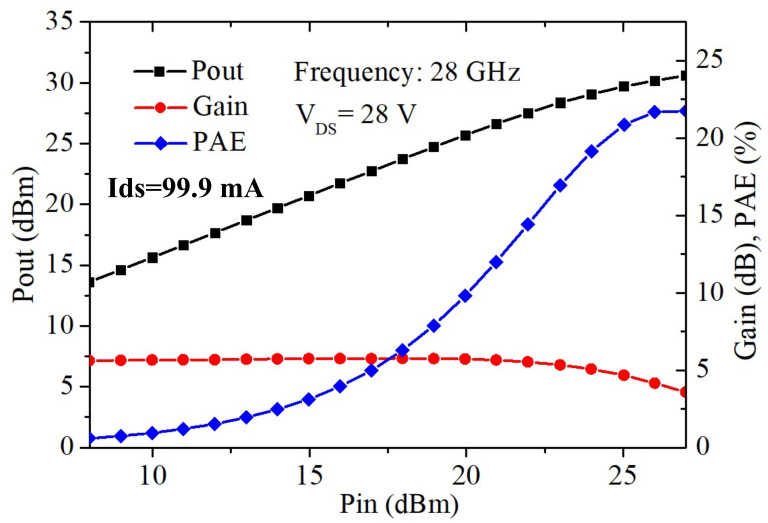
Power performance at 28 GHz for the 8 × 50 μm GaN:C/EBL buffer device with V_DS_ = 28 V.

On-wafer load-pull measurement for the device with large gate peripheries of 8 × 50 μm at 28 GHz were performed: Γsource tuning of the device for max Gain and Γload tuning for max Power. The measurement results of the device at V_DS_ = 28 V are shown in Figure 6. The device with the GaN:C/EBL buffer exhibited a high Pout of 31.2 dBm, with a power density over 3 W/mm and PAE of 21.7% at V_DS_ = 28 V; these results demonstrate the GaN:C/EBL buffer has a high breakdown field strength, and can reduce the hot electron trapping effect even at high V_DS_ when operated at high frequencies.

## 4. Conclusions

The AlGaN/GaN HEMTs on Si substrate using the GaN:C buffer with AlGaN EBL is fabricated and evaluated for millimeter-wave applications. Compared with the DHFET, the GaN:C/EBL buffer device has a higher breakdown strength, higher thermal stability and better RF performances. This is because the GaN:C buffer can reduce the leakage current, and the insertion of an AlGaN EBL in the GaN:C buffer can prevent carbon proximity effects from the buffer for the device. An output power of 31.2 dBm with a PAE of 21.7% was observed at 28 GHz when the drain was bias at 28 V for the 8×50 μm GaN:C/EBL buffer device. The results demonstrate that the AlGaN/GaN HEMTs on Si with GaN:C/EBL buffer is suitable for high-frequency device applications up to millimeter-wave. In the future, we will be using the GaN:C buffer with different AlGaN EBLs to achieve a GaN device for V band applications.

## Data Availability

The data presented in this paper are available on request from the corresponding author.
